# Occupation-Associated Fatal Limbic Encephalitis Caused by Variegated Squirrel Bornavirus 1, Germany, 2013

**DOI:** 10.3201/eid2406.172027

**Published:** 2018-06

**Authors:** Dennis Tappe, Kore Schlottau, Daniel Cadar, Bernd Hoffmann, Lorenz Balke, Burkhard Bewig, Donata Hoffmann, Philip Eisermann, Helmut Fickenscher, Andi Krumbholz, Helmut Laufs, Monika Huhndorf, Maria Rosenthal, Walter Schulz-Schaeffer, Gabriele Ismer, Sven-Kevin Hotop, Mark Brönstrup, Anthonina Ott, Jonas Schmidt-Chanasit, Martin Beer

**Affiliations:** Bernhard Nocht Institute for Tropical Medicine, Hamburg, Germany (D. Tappe, D. Cadar, P. Eisermann, M. Rosenthal, J. Schmidt-Chanasit);; Friedrich-Loeffler-Institut, Greifswald-Insel Riems, Germany (K. Schlottau, B. Hoffmann, D. Hoffmann, M. Beer);; University Medical Center Schleswig-Holstein, Kiel, Germany (L. Balke, B. Bewig, H. Laufs, M. Huhndorf);; Christian-Albrecht University of Kiel and University Medical Center, Kiel (H. Fickenscher, A. Krumbholz);; Saarland University Medical Center, Homburg, Germany (W. Schulz-Schaeffer);; Zoological Garden, Schleswig-Holstein, Germany (G. Ismer);; Helmholtz Centre for Infection Research and German Centre for Infection Research, Braunschweig, Germany (S.-K. Hotop, M. Brönstrup);; Euroimmun AG, Lübeck, Germany (A. Ott);; German Centre for Infection Research, Hamburg (J. Schmidt-Chanasit)

**Keywords:** Bornavirus, VSBV-1, limbic encephalitis, occupational risk, transmission, viruses, squirrel, Germany, variegated squirrel bornavirus 1, zoonoses

## Abstract

Limbic encephalitis is commonly regarded as an autoimmune-mediated disease. However, after the recent detection of zoonotic variegated squirrel bornavirus 1 in a Prevost’s squirrel (*Callosciurus prevostii*) in a zoo in northern Germany, we retrospectively investigated a fatal case in an autoantibody-seronegative animal caretaker who had worked at that zoo. The virus had been discovered in 2015 as the cause of a cluster of cases of fatal encephalitis among breeders of variegated squirrels (*Sciurus variegatoides*) in eastern Germany. Molecular assays and immunohistochemistry detected a limbic distribution of the virus in brain tissue of the animal caretaker. Phylogenetic analyses demonstrated a spillover infection from the Prevost’s squirrel. Antibodies against bornaviruses were detected in the patient’s cerebrospinal fluid by immunofluorescence and newly developed ELISAs and immunoblot. The putative antigenic epitope was identified on the viral nucleoprotein. Other zoo workers were not infected; however, avoidance of direct contact with exotic squirrels and screening of squirrels are recommended.

## Abstract 2

This case underscores the risk for spillover infections to humans who work with exotic squirrels.

Limbic encephalitis, a term coined in 1960 as a clinical/anatomic description (*1*), is a rare regional inflammation of the brain involving mainly the limbic system but also other anatomic structures. Clinically, limbic encephalitis onset is subacute, and the disease is characterized by short-term memory deficits, seizures, and psychiatric symptoms (*2*). The disease is commonly regarded as an autoimmune-mediated condition associated with autoantibodies directed against various intracellular or neuronal cell surface/synaptic antigens induced by underlying neoplasia, such as thymoma or small cell lung cancer (*2*–*4*). However, several cases of unknown etiology in patients seronegative for autoantibodies have been reported (*5*–*8*).

We retrospectively investigated a case of unexplained fatal limbic encephalitis in a seronegative animal caretaker at a zoological garden in northern Germany in 2013. Our investigation was triggered by the recent detection of the novel zoonotic variegated squirrel bornavirus 1 (VSBV-1) by real-time reverse transcription PCR (rRT-PCR) in an exotic Southeast Asian Prevost’s squirrel (*Callosciurus prevostii*) from the zoo where the caretaker had worked (*9*). VSBV-1 had been discovered in 2015 as the cause of a cluster of fatal cases of encephalitis in eastern Germany among private breeders of another exotic squirrel species, the Central American variegated squirrel (*Sciurus variegatoides*) (*10*). In addition to investigating the fatal encephalitis case, we used newly developed tests for VSBV-1 to serologically screen all animal caretakers at the zoo who had contact with squirrels.

## Patient, Materials, and Methods

### The Case 

**Figure 1:**
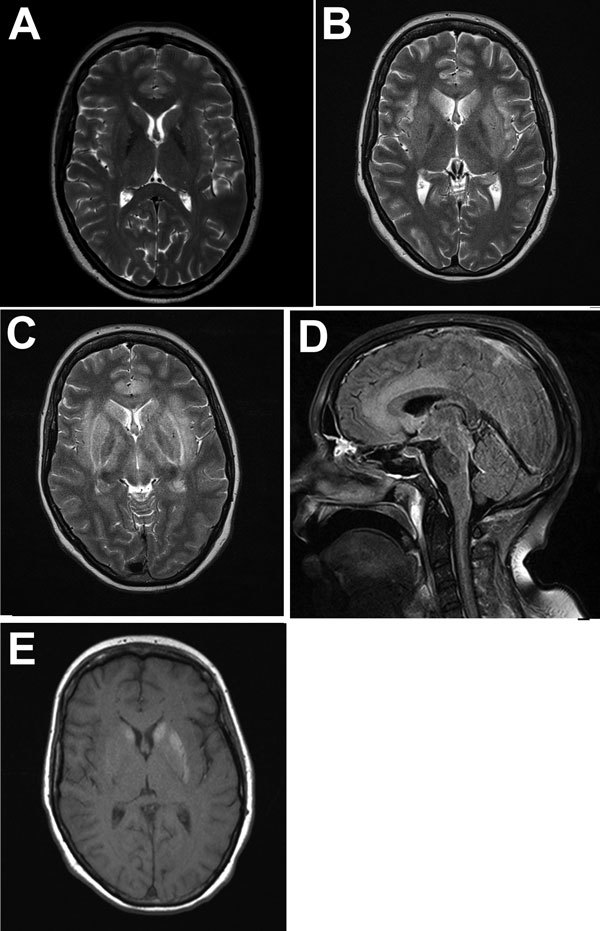
Magnetic resonance imaging of the brain throughout the course of the disease in patient who died of limbic encephalitis caused by variegated squirrel bornavirus 1 (VSBV-1), Germany, 2013. A) T2-weighted transversal image at admission showing no pathologic changes. B) T2-weighted image 3 weeks after admission showing edema in limbic structures (insula, hippocampus, anterior cingulate) and in the basal ganglia. C) T2-weighted image 4 weeks after admission showing progressive edema. Additional myelopathy extended from the medulla down into the thoracic segments (not shown). D) FLAIR image 4 weeks after admission showing edema in the anterior cingulate cortex. E) T1-weighted image 4 weeks after admission without contrast showing slight hemorrhage in the basal ganglia.

In July 2013, a 45-year-old female zoo animal caretaker from the federal state of Schleswig-Holstein, northern Germany, experienced fever, dysphonia, cough, pharyngitis, vertigo, and paresthesia (below her eye), followed by ataxia, coma, and pituitary gland insufficiency. The patient had had no previous medical conditions and no history of immunosuppression, and her HIV serologic results had been negative. Cerebrospinal fluid (CSF) analysis showed lymphocytic pleocytosis. Peripheral blood inflammatory parameters were elevated, with relative neutrophilia and lymphopenia. Initial cranial magnetic resonance images (MRIs) showed no abnormalities. Follow-up MRIs taken 3 weeks later demonstrated lesions in a bilateral limbic distribution (medial temporal lobes, anterior cingulum, insula, hippocampus, hypothalamus, periventricular tectum), in the basal ganglia (Figure 1), and in the upper myelon. Limbic encephalitis was diagnosed on the basis of morphologic appearance and progressed within 1 week. Extended laboratory workup results for central nervous system (CNS) infection and autoimmune disease were within reference limits (Technical Appendix Table 1). No underlying neoplasia was detected. Repeated electroencephalography showed generalized slow activity and evidence of a current nonconvulsive epileptic seizure. Histopathologic examination of a brain biopsy sample had demonstrated glial activation and lymphocyte infiltration. No neurotropic bacteria, fungi, parasites, or viruses had been detected by microscopy, culture, or PCR. Creutzfeldt-Jakob disease was excluded by protein aggregate filtration and paraffin-embedded tissue blotting (*11*). The patient had required mechanical ventilation because of bilateral pneumonia and received broad antiinfective chemotherapy (including acyclovir throughout), anticonvulsants, and steroids later in the course of the disease (Technical Appendix Table 1). Within 3 months after symptom onset, she died of myeloencephalitis of undetermined etiology. Postmortem examination of the brain demonstrated edema, necrosis, and perivascular lymphocyte cuffing in limbic structures and in the basal ganglia.

### Molecular Investigations

Patient samples available for analysis for this study were archived frozen CSF and formalin-fixed paraffin-embedded brain tissue, myocardium, lungs, kidney, liver, spleen, pancreas, bone marrow, and intestine. We performed VSBV-1–specific rRT-PCR on all samples as described (*10*), using a detection limit of 10 genome equivalents/reaction. 

Metagenomic analysis of the brain samples followed a standard workflow as described (*12*,*13*). We used a MiSeq instrument (Illumina, San Diego, CA, USA) for sequencing, or we performed Sanger sequencing as described (*9*). VSBV-1 sequences from the animal caretaker and 4 species of squirrel (*Callosciurus prevostii*, *C. finlaysonii*, *Sciurus variegatoides*, and *Tamiops swinhoei*) from different zoos and other holdings were compared with previously described VSBV-1 sequences. Comparisons were performed by the Bayesian Monte Carlo Markov Chain sampling method implemented in BEAST (*14*) and in parallel a maximum-likelihood inference by using PhyML version 3.1 (*15*) with the complete genome and complete major protein N (p40) gene sequences. Using the Akaike information criterion in jModelTest 2 (*16*), we found that the best nucleotide substitution model that fit the data was the general time-reversible plus gamma plus invariate sites model. 

We aligned sequences by using the MAFFT (multiple alignment using fast Fourier transform) algorithm and compared and analyzed the VSBV-1 genomes in Geneious version 9.1.4 (https://www.geneious.com/). All sequences were confirmed as nonrecombinant by the various methods for recombination detection implemented in RDP4 (*17*).

### Immunohistochemistry

We obtained polyclonal antiserum against VSBV-1 and Borna disease virus (BoDV) N and P proteins from rabbits immunized with the respective recombinant antigens and purified by protein A ion exchange (Davids Biotechnologie, Regensburg, Germany). We tested reactivity of the rabbit antiserum and preimmune serum by immunofluorescence antibody test (IFAT) and on a newly developed bornavirus immunoblot. We used 10 unrelated human brain tissue samples as negative controls. After pretreatment with proteinase K and endogenous peroxidase blocking, we incubated the formalin-fixed paraffin-embedded sections with the antiserum (1:1,000–1:5,000 in phosphate-buffered saline at room temperature overnight), followed by a goat anti-rabbit biotinylated polymer antibody, a streptavidin-horseradish peroxidase complex, and the 3-amino-9-ethylcarbazole substrate (DCS, Hamburg, Germany).

### Serologic Assays and Antibody Epitope Mapping

To detect bornavirus-specific IgG in serum and CSF, we used a persistently BoDV-infected cell line in a standard indirect IFAT (*10*); we also developed an ELISA and an immunoblot. We screened protein A–purified antibodies from the patient’s CSF by using peptide microarrays as described (*18*). For the VSBV-1 IgG ELISA, sequences of VSBV-1 N and P genes were cloned and expressed as maltose-binding protein (MBP) fusion proteins in *Escherichia coli* strain BL21 Gold (DE3) (Novagen-Merck, Darmstadt, Germany). The protein was purified by amylose affinity chromatography and eluted. The N terminal MBP tag was cleaved by a 3C protease at 4°C overnight. After the protease was removed, we further purified the protein sample by size exclusion chromatography (Superdex 200, Sigma-Aldrich, Munich, Germany). We coated polystyrene microtiter plates (Polysorp; Nunc, Roskilde, Denmark) with 2 μg/mL VSBV-1 N or P protein and incubated them overnight at 4°C. After blocking with 6% bovine serum albumin, we added 1:400 diluted human serum in 1% bovine serum albumin diluent. After incubation for 2 h at 37°C, we added 100 μL anti-human IgG (Dako Cytomation, Hamburg, Germany; diluted 1:6,000) and incubated the plates at 37°C for 1 h. The reaction was stopped after 5 min incubation at room temperature with 3,3′,5,5′-tetramethylbenzidine. Last, we measured the optical density (OD) at 450 nm (reference 620 nm) and calculated the final OD for each serum sample as the difference between the OD measured in VSBV-1 N- or P-containing and MBP-containing wells. The final ODs for serum dilutions of 1:400 were regarded as positive if the mean OD exceeded the mean OD + 3 SD obtained with 200 control samples from healthy blood donors. For the bornavirus IgG immunoblot (line immunoassay system; EUROIMMUN AG, Lübeck, Germany), we coated recombinantly expressed VSBV-1 and BoDV N and P proteins on a nitrocellulose membrane as narrow lines. We incubated the strips at room temperature with serum (30 min, 1:50) or CSF (3 h, 1:4), followed by alkaline phosphatase-conjugate (serum 30 min, CSF 1 h; 1:10 each) and nitro-blue-tetrazoliumchloride as substrate (serum 10 min, CSF 20 min). Intensities of the detected antibodies were automatically evaluated by using the EUROLineScan software (EUROIMMUN AG). For validation, we tested 150 samples from healthy blood donors from northern Germany. For the antibody epitope mapping, we synthesized 360 15-mer peptides covering the whole p40 sequence of VSBV-1 (GenBank accession no. CEK41887) with an offset of 1 aa by using the synthetic peptide arrays on membrane support technique (*19*) and printed them onto glass slides by using the SC2 method (*20*). We also performed an alanine scan for highest antibody binding. Slides were washed with absolute ethanol for 3 min and then 3 times with Tris-buffered saline (TBS) for 3 min. Next, we blocked slides overnight at room temperature with 2% (wt/vol) casein in TBS-Tween 20. The CSF sample was diluted 1:120 in blocking buffer. We incubated the slides with the sample overnight in a humidified chamber at 4°C. After washing 3 times (5 min) in TBS-Tween 20, we incubated the slides with anti-human IgG coupled to Cy5 for visualization of bound antibodies at a dilution of 1:240 in blocking buffer for 1.5 h at room temperature. After washing slides twice in TBS-Tween 20 followed by 3 times in distilled water (5 min) and dried in a nitrogen stream, we immediately scanned slides by using an Agilent DNA Microarray scanner (Agilent Technologies, Waldbronn, Germany). The resulting pictures were obtained by using Agilent Feature Extraction Software version 10.7.3. We used the program Chimera (*21*) for 3D illustrations and distance measurements.

## Results

### rRT-PCR Detection, Whole-Genome Sequence, and Phylogeny of VSBV-1 

**Figure 2:**
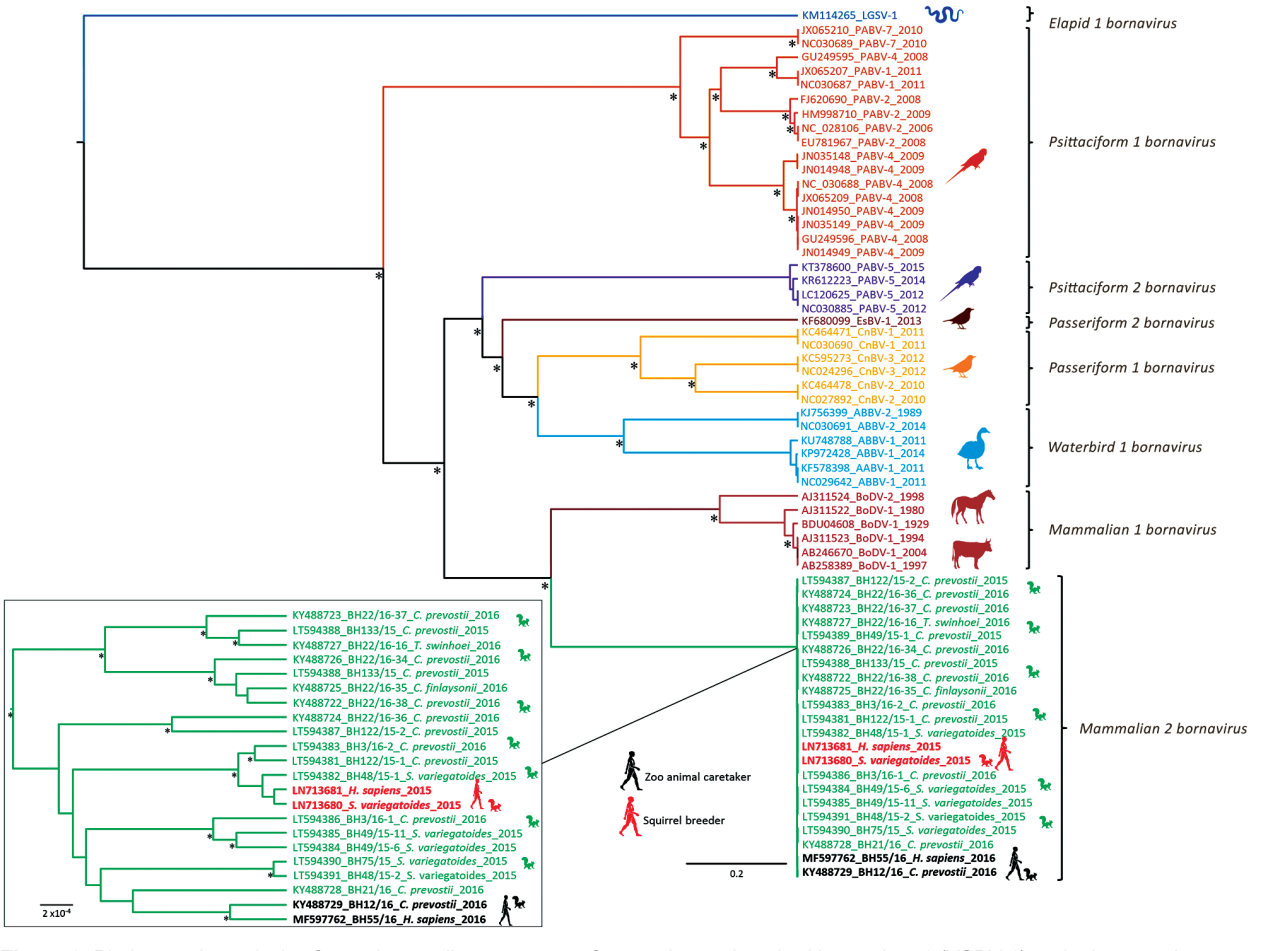
Phylogenetic analysis of complete coding sequences from variegated squirrel bornavirus 1 (VSBV-1) and other members of *Bornaviridae*. The phylogenetic trees were inferred by using the Bayesian Markov Chain Monte Carlo method and in parallel a maximum-likelihood method (tree not shown). Statistical support of grouping from Bayesian posterior probabilities (clade credibilities >90%) and maximum-likelihood bootstrap replicates (>70%) are indicated with an asterisk. Taxon information includes GenBank accession number and virus abbreviation. Branch colors are based on bornavirus species. The VSBV-1 sequence from the patient generated during this study and the highly similar VSBV-1 sequence from the zoo squirrel are shown in bold. Inset shows detail of mammalian 2 bornavirus section. Scale bars represent nucleotide substitutions per site.

We detected VSBV-1 RNA in the patient’s CSF, plexus, paraventricular brain areas, striatum, substantia nigra, and cerebellum (quantitation cycle values 24.1–35.2), whereas PCR of 8 cortical areas was negative (Technical Appendix Table 2). PCR of myocardium, lung, liver, kidney, spleen, bone marrow, pancreas, and intestine samples also yielded negative results. Phylogenetic analysis of the complete genome (Figure 2) and the N protein gene datasets (Technical Appendix Figure 1) demonstrated that the newly sequenced VSBV-1 from the deceased animal caretaker and previously described VSBV-1 strains from the exotic squirrels formed a distinct and highly supported monophyletic group (novel species *Mammalian 2 bornavirus*) within the bornavirus phylogeny. Phylogenetic analysis performed only on the VSBV-1 dataset revealed that the VSBV-1 strain from the animal caretaker (BH55/16) clustered with the virus sequence from the single infected contact *C. prevostii* squirrel (BH12/16) from the zoo (Figure 2, inset; Technical Appendix Figure 1, inset). Identity matrix analysis showed 99.90% nt and 99.92% aa identity between the BH55/16 and BH12/16 VSBV-1 strains; identities with other VSBV-1 strains were 99.69%–99.80% nt for BH55/16 and 98.41%–99.40% aa for BH12/16. A single unique amino acid substitution (N_390_T) was found in the G gene of both strains (Table 1), and 3 unique synonymous nucleotide substitutions were detected in the N, M, and G genes.

**Table 1:** Comparison of amino acid substitutions of human-derived variegated squirrel bornavirus 1 strain from a deceased zoo animal caretaker with those detected in exotic squirrels of 3 species and private squirrel breeders on the basis of protein coding region sequences*

Protein	Human (*Homo sapiens*) BH55/16	Squirrel			Unique substitutions
		*Sciurus variegatoides*	*Callosciurus prevostii*	*Tamiops swinhoei*	
N	No	No	No	No	0/0
X	No	No	**T_120_I**	No	1/1
P	No	L_93_I	**P_21_S**, L_93_I	L_93_I	1/2
M	No	No	**S_71_F**	No	1/1
G	**S_175_N**, **E_185_D**,V_228_M, **N_390_T**	S_59_P, **E_73_K**, **S_165_G**, T_170_A, **N_188_D**, V_228_M, S_237_P, **R_244_K**, **V_313_I**, **N_390_S**	**S_19_L**, **E_60_K**, **A_106_V**, **N_111_D**, T_170_A, **S_175_N/I**, **N_188_S**, **G_196_D**, V_228_M, S_237_P, S_238_L, **S_254_P**, **S_332_P**, **Y_335_S**, **N_390_T**, **I_480_M**	S_59_P, S_237_P, S_238_L	17/24
L	I_112_V, L_779_F, T_832_I, G_1365_R, G_1388_S	I_112_V, **E_143_K**, M_166_V, S_360_L, A_756_V, L_779_F, T_832_I, G_1365_R, G_1388_S	**K_63_R**, I_112_V, **N_136_S**, **A_144_T**, M_166_V, **V_539_I**, A_756_V, L_779_F, T_832_I, **I_1053_V**, **K_1170_L/R**, **Q_1286_H**, G_1365_R, G_1388_S, **P_1423_A**, **S_1474_L**	M_166_V, **I_194_V**, S_360_L	11/19

*Boldface indicates the unique amino acid substitutions detected in each host. Boldface and underlining indicate the mutation detected only in the variegated squirrel bornavirus 1 strain from the deceased animal caretaker (BH55/16; GenBank accession no. MF597762) and her contact squirrel (*Callosciurus prevosti*)*.*

### Immunohistochemical Findings

**Figure 3:**
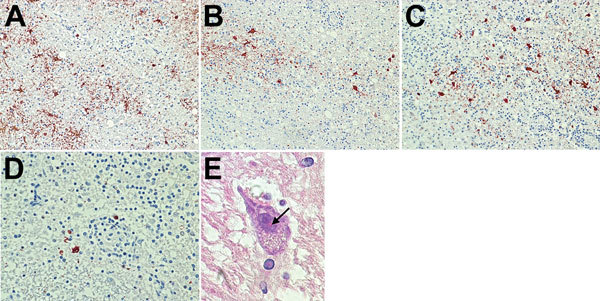
Immunohistochemical and histologic slides of brain of patient who died of limbic encephalitis caused by variegated squirrel bornavirus 1 (VSBV-1), Germany, 2013. Immunohistochemistry of viral antigen in subcortical areas of the brain was performed by using a polyclonal antiserum against VSBV-1 N protein. Viral antigen was present in neurons and glial cells in nuclei and cytoplasm. A) Substantia nigra. Immunoperoxidase stain with hematoxylin counterstain; original magnification ×200. B) Striatum. Immunoperoxidase stain with hematoxylin counterstain; original magnification ×200. C) Subcortical area next to the hypothalamus. Immunoperoxidase stain with hematoxylin counterstain; original magnification ×200. D) Subcortical area next to the hippocampus. Immunoperoxidase stain with hematoxylin counterstain; original magnification ×400. E) Intranuclear eosinophilic inclusion body resembling a bornavirus-like Joest-Degen body (arrow). Hematoxylin and eosin stain; original magnification ×600.

When we applied the VSBV-1 and BoDV N protein antiserum, brain tissue sections of the subcortical areas showed nuclear and neuropil immunostaining of brain cells (Figure 3). All sections of uninfected human control brain were negative. The rabbit antiserum showed a nuclear pattern by IFAT (endpoint titer for BoDV N 10,240) and strong reactivity with respective homologous and heterologous antigens on the immunoblot. The respective preimmune serum samples were negative by IFAT, immunoblot, and immunohistochemistry. In addition, intranuclear eosinophilic inclusion bodies, resembling bornavirus-like Joest-Degen bodies, were visible during histopathologic examination of the basal ganglia (Figure 3).

### Serology and Antibody Epitope Mapping Findings 

**Figure 4:**
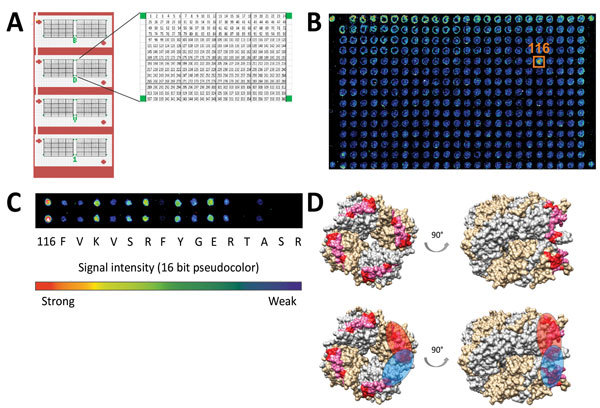
N protein (p40) peptide microarray–based epitope mapping of variegated squirrel bornavirus 1 from patient who died of limbic encephalitis, Germany, 2013. A) The N protein–based peptide microarray chip consists of 8 identical arrays composed of 360 15-mer peptides with an offset of 1 aa. Each subarray was bordered by biotin spots (green). B) Representative single-channel readouts from 1 subarray in 16-bit pseudocolor is given for the protein A–purified patient cerebrospinal fluid sample. Signals were classified positive if they appeared in each of the 8 subarrays. The detected signal is surrounded by an orange box and labeled by the corresponding spot number. C) The alanine scan of spot 116 showed highest antibody binding for the original sequence and less bound antibodies after substitution of the indicated amino acids by alanine. D) Mapping of antibody target sequences onto the tetrameric P40 nucleoprotein 3D-structure (PDB:1N93). Monomers are depicted in gray and gold, amino acids necessary for antibody binding (red) and inconsequential (pink) related to spot 116. Potential binding sites of antibodies for spot 116 are shown in the lower panels as red (1 monomer) and blue (spanning 2 adjacent monomers and forming a binding pocket) overlay.

We detected bornavirus-specific IgG in high concentrations in the patient’s CSF by IFAT in a nuclear pattern (IgG endpoint titer 2,560 [Technical Appendix Figure 2]) and by ELISA (OD 1.41 against N protein and 0.30 against VSBV-1 P protein). IgG reactivity on the immunoblot against VSBV-1 N was strong and against VSBV-1 P and BoDV P antigens was less (Table 2). Patient serum was no longer available for testing. Epitope mapping of the protein A–purified CSF antibodies revealed a single spot signal on the peptide microarray corresponding to the sequence 116-FVKVSRFYGERTASR-130 (Figure 4). An alanine scan array of the detected peptide showed that 8 aa of the 15-mer peptide were found to be essential for antibody binding at the N-terminus and particularly at the C-terminus of the peptide sequence. Mapping of the epitope to the 3D-structure 1N93 of the viral p40 nucleoprotein showed that the peptide is part of the N terminal accessible surface. The measured distances of <25Å of the covered area suggest that the antibody target sequences form a discontinuous epitope or are located on 2 adjacent monomers because the N terminal part of the epitope FVXV and the C-terminal part TASR form a binding pocket with a distance of 12Å.

**Table 2:** Serologic test results for zoo animal caretaker who died of encephalitis, other zoo animal caretakers, and healthy blood donors, Germany, 2013*

Patient or group, sample type	IgG immunoblot†				BoDV IgG IFAT	VSBV-1 IgG ELISA‡		Contact with Prevost's squirrels
BoDV-P	BoDV-N	VSBV-P	VSBV-N	VSBV-P	VSBV-N
Encephalitis patient, age, y/sex, CSF								
45 y/F	**+**	–	**+**	**+**	**1:2,560**	Pos	Pos	Regularly
14 zoo animal caretakers, age, y/sex, serum								
44 y/F	–	–	–	**(+)**	Neg	Neg	Neg	Regularly
32 y/M	–	–	–	–	Neg	Neg	Neg	Regularly
25 y/F	–	**+**	–	**(+)**	Neg	Neg	Neg	Rarely
33 y/F	–	–	–	–	Neg	Neg	Neg	Occasionally
26 y/F	–	–	–	**(+)**	**1:160§**	Neg	Neg	Occasionally
27 y/M	–	**+**	–	–	Neg	Neg	Neg	Occasionally
29 y/F	–	–	–	–	Neg	Neg	Neg	Rarely
48 y/F	–	**(+)**	–	**+**	**1:40**	Neg	Neg	Rarely
35 y/M	–	–	–	–	Neg	Neg	Neg	Occasionally
24 y/F	–	–	–	–	Neg	Neg	Neg	Regularly
18 y/F	–	**+**	–	–	Neg	Neg	Neg	Regularly
21 y/F	–	–	–	**(+)**	Neg	Neg	Neg	Occasionally
37 y/M	–	–	–	**(+)**	Neg	Neg	Neg	Regularly
20 y/F	–	–	–	–	Neg	Neg	Neg	Regularly
150 healthy blood donors, %, serum								
Positive	1.5	0	1.5	1.5	NA	Neg	Neg	No
Weakly positive	4.5	4.5	0	12	NA	Neg	Neg	No

*Boldface indicates positive response. BoDV, borna disease virus; CSF, cerebrospinal fluid; ELISA; enzyme-linked immunosorbent assay; IFAT, immunofluorescence antibody test; NA, not available; neg, negative; OD, optical density; pos, positive; VSBV-1, variegated squirrel bornavirus 1;. +, strongly positive; (+), weakly positive**;** –, no reaction by immunoblot.  †Results were obtained densitometrically using EUROLineScan software (EUROIMMUN AG, Lübeck, Germany) and transformed into semiquantitative categories (negative, –, 0–13; weakly positive, (+), 14–20; positive, +, 21–255). ‡Final OD values for serum dilutions of 1:400 were regarded as positive if the mean OD exceeded the mean OD + 3 SD obtained with negative control samples. §The IgG endpoint titer is defined as the reciprocal of the highest analyte dilution that gives a positive signal in IFAT.

### Serologic Screening Results for Zoo Animal Caretakers

Serum samples from all 14 animal caretakers were analyzed by ELISA, IFAT, and immunoblot. Of these 14 caretakers, 6 had regular contact with the squirrels (feeding, cleaning), 5 had occasional contact, and 3 had only rare contact. By immunoblot, of the 14 samples, 6 showed a positive or weakly positive reaction with VSBV-1 N and 4 with BoDV N but not with the respective P antigens. IFAT showed low titers for 2 of the 14 samples, whereas ELISA results were negative (Table 2). Serologic reactivity did not correlate with the reported intensity of squirrel contact. By immunoblot, samples from 13.5% of blood donors showed a positive or weakly positive reaction with VSBV-1 N, 4.5% with BoDV N, 1.5% with VSBV-1 P, and 6% with BoDV P antigens (Table 2). None of the blood donor samples reacted against >1 bornavirus antigen.

## Discussion

The association of a fatal case of encephalitis with the recently discovered zoonotic VSBV-1 was achieved by molecular assays, specific immunohistochemistry, and epitope-resolved serology. We found Joest-Degen inclusion bodies typical for a bornavirus infection in the brain tissue of the human patient. Genetic characterization and phylogenetic tree reconstruction of the VSBV-1 sequence from the patient demonstrated spillover infection originating from the infected Prevost’s squirrel from the zoologic garden. This spillover hypothesis is supported by our finding of a unique amino acid substitution (N_390_T) in the G gene and 3 unique synonymous nucleotide substitutions of VSBV-1 in both strains. The patient was therefore most likely infected during her work with the squirrels in the zoo. VSBV-1–infected *Sciuridae* squirrels show only mild inflammation of the brain and no clinical symptoms (*10*,*22*) and are therefore probably a natural reservoir for VSBV-1. In addition to virus presence in the brain of exotic squirrels with confirmed VSBV-1 infection, high viral genome loads were detected in skin, salivary glands, kidneys, and urinary bladder (*22*). The virus is probably transmitted by scratches and bites (*10*); however, infective viral particles have yet to be demonstrated in animal secretions or skin scalings. 

The origin of this virus is unknown. Squirrel species from 2 subfamilies in different holdings in Europe were infected: Central America squirrel species (subfamily *Sciurinae*: *S. variegatoides* and *S. granatensis*) and Southeast Asia squirrel species (subfamily *Callosciurinae*: *C. prevostii*, *C. finlaysonii*, and *T. swinhoei*) (*9*). Phylogenetic analysis of the complete genome and complete p40 gene from VSBV-1 from the animal caretaker and comparison with sequences from previously identified infected exotic squirrel species and squirrel breeders further strengthen our previous reports (*9*,*10*,*22*) that the VSBV-1 strains form a distinct and highly divergent monophyletic lineage within the bornavirus phylogeny.

The case we describe contrasts in several aspects with the previously reported cluster of fatal encephalitis cases in 3 elderly male private squirrel breeders in eastern Germany (*10*), which were associated with Central America variegated squirrel species. The infection we report emerged as an occupation-associated disease in an animal caretaker in a zoo in northern Germany and was linked to a different species of exotic squirrel, a Southeast Asia Prevost’s squirrel. Moreover, the patient we report was female, <60 years of age, and had no medical preconditions. The myoclonus and thrombosis reported for the squirrel breeders (*10*) was absent in the zoo worker. However, the fever, ataxia, coma, duration of illness (subacute-onset encephalitis), and late appearance of MRI changes in the zoo worker are similar to findings in the previous cluster. In the case we describe, VSBV-1 in the central nervous system of the zoo worker was in a limbic distribution. In a rat model, the related BoDV has a preferential limbic tropism (*23*). Limbic encephalitis in humans is regarded as a regional autoimmune encephalitis that predominantly affects the limbic system. The disease is associated with paraneoplastic (epiphenomenal) nonpathogenic autoantibodies directed against intracellular epitopes (Hu, Ri, Ma2, and GAD), and pathogenic neuronal cell surface autoantibodies (against AMPA, GABA, and voltage-gated potassium-receptor complexes) (*2*–*4*), none of which were detected in the zoo worker. Recently, the triggering of an autoantibody-positive, nonparaneoplastic limbic encephalitis by human herpesvirus 6B was speculated (*24*), similar to the induction of NMDA (N-methyl-D-aspartate) receptor encephalitis as clinical relapse after herpes simplex virus encephalitis (*25*). Of note, several cases of limbic encephalitis without autoantibodies and with unknown etiology have been reported (*5*–*8*). Hypothesizing that the pathogenesis of such seronegative limbic encephalitis cases without neoplasia is infectious or postinfectious remains tempting. In concordance with limbic encephalitis, for the patient reported here, electroencephalogram changes demonstrated epileptic activity, histologic examination showed lymphocyte involvement, and CSF showed a lymphocytic pleocytosis with raised protein levels (*2*,*5*). Treatment for seronegative limbic encephalitis thus far comprises immunomodulatory drugs, including intravenous immunoglobulins (*5*–*7*).

No curative treatment has been established for human bornavirus infections. Ribavirin, which has shown in vitro effectiveness against various bornaviruses (*26*–*28*), was not administered to the patient we describe. In the VSBV-1 encephalitis cluster among squirrel breeders (*10*), ribavirin had been given to 1 person but had no clinical effect. In animals, BoDV pathology is caused by the host’s immune response (*29*–*31*). Whether an immunosuppressive treatment, as administered here and previously to 2 squirrel breeders (*10*), is clinically beneficial for patients with bornavirus infections remains unknown.

We developed novel serologic assays that showed a good correlation of ELISA, IFAT, and immunoblot results for the encephalitis patient. IFAT and immunoblot showed serologic cross-reactivity against BoDV antigens. In the patient’s CSF sample, a single spot reaction to the viral N protein could be demonstrated by peptide microarray. We were able to project the epitope onto the surface of the 1N93 3D-structure. In the small number of healthy animal caretakers tested (14), the constellation of antibody reactivity was unclear and inconsistent. Whether the weak immunoblot and IFA antibody responses against bornavirus antigens in 2 healthy animal caretakers reflect a past contact with VSBV-1 remains speculative. A low percentage of positive reactions to single bornavirus antigens was detected by the immunoblot in healthy blood donors. This finding is probably nonspecific because none of the healthy persons screened in our study exhibited the antibody constellation that was seen in the confirmed VSBV-1–infected patient. Future studies, including T-cell assays, will address the immunologic response in humans in more detail. More seroprevalence studies of predominantly zoo animal caretakers and squirrel breeders are under way to evaluate the serologic tests and shed more light on the human exposure to VSBV-1.

Our investigation highlights the risk for VSBV-1 transmission from zoo animals to humans, especially in view of the previously described relatively high rate of infection among squirrels of these 2 families (*Callosciurinae,* 8.5%; *Sciurinae,*1.5% [*9*]). These findings further emphasize the need to test all exotic squirrels for VSBV-1 to prevent further spillover infections. Reasonable precautions should be taken, such as having zoo employees, zoo visitors, and private breeders avoid direct contact with exotic squirrels. Moreover, for patients with signs of limbic encephalitis without underlying autoimmunopathology (seronegative limbic encephalitis), differential diagnostics should be adapted, possible infection with VSBV-1 should be investigated, and patients should be asked whether they have had contact with exotic squirrels. According to results of in vitro studies with other bornaviruses (*26*–*28*), early and extended treatment with ribavirin might be considered for humans with VSBV-1 infection.

Technical Appendix Supplementary results for study of case of occupation-associated fatal limbic encephalitis caused by variegated squirrel bornavirus 1, Germany, 2013.
