# Supplementary material for: Occupation-Associated Fatal Limbic Encephalitis Caused by Variegated Squirrel Bornavirus 1, Germany, 2013
Source: Emerg Infect Dis. 2018 Jun;24(6):978–87. doi: 10.3201/eid2406.172027 (PMC6004865; doi:10.3201/eid2406.172027)
Supplement: Technical Appendix — Supplementary results for study of case of occupation-associated fatal limbic encephalitis caused by variegated squirrel bornavirus 1, Germany, 2013. [file 17-2027-Techapp-s1.pdf]

Article DOI: https://doi.org/10.3201/eid2406.172027

Occupation-Associated Fatal Limbic Encephalitis Caused by Variegated Squirrel Bornavirus 1, Germany, 2013

Technical Appendix

Technical Appendix Table 1. Results of laboratory analyses and patient treatment details*

Analysis or treatment	Description
Initial peripheral blood inflammatory parameters	Increased procalcitonin concentration (0.44 ng/mL, normal <0.05 ng/mL), with normal CRP levels; normal leukocyte count 9.17 Gpt/L with relative neutrophilia (82.1%) and relative lymphopenia (9.1%)
Initial CSF analyses	157 cells/µL (normal <5/µL; mostly lymphocytes), total protein 1832 mg/L (normal <500 mg/L), lactate 4.4 mmol/L (normal <2.1 mmol/L), tau-protein 2750 pg/mL (normal 100–300 pg/mL), normal glucose levels
Negative pathogen serology†	West Nile virus, tick-borne encephalitis virus, lymphocytic choriomeningitis virus, human immunodeficiency virus, hepatitis B and C virus, rabies virus, hantaviruses; *Treponema pallidum, Borrelia burgdorferi*, *Leptospira* spp., *Chlamydophila psittaci, Rickettsia typhi, Brucella* spp.*, Coxiella burnettii, Mycoplasma pneumoniae; Toxoplasma gondii*
Negative pathogen serology CSF	West Nile virus, tick-borne encephalitis virus, lymphocytic choriomeningitis virus; *Treponema pallidum, Borrelia burgdorferi, Aspergillus*, *Candida*, and *Cryptococcus* antigen
Negative molecular pathogen examinations CSF	Rabies virus, herpesviruses (cytomegalovirus, Epstein-Barr virus, herpes simplex virus, varicella-zoster virus, human herpes virus 6, simian herpes virus B), human immunodeficiency virus, tick-borne encephalitis virus, enteroviruses; *Borrelia burgdorferi*, eubacterial and panfungal PCRs
Negative molecular pathogen examinations biopsy	Mumps virus, measles virus, rubella virus, enteroviruses; eubacterial and panfungal PCRs, *Balamuthia mandrillaris, Naegleria fowleri*
Negative autoimmune serology	ANA, p-ANCA, c-ANCA, cardiolipin, Hu (ANNA-1), ANNA-3, Ma/Ta, AMPA, GABA B-receptor, GAD, NMDA receptor, VGKC, LGI1
Negative autoimmune serology CSF‡	Hu (ANNA-1), ANNA-3, Ma/Ta, CV2, ganglioside GQ1b, NMDA receptor, Yo, Ri, titin, Zic4, tr/DNER, SOX1, amphiphysin
Anti-infective treatment	Ceftriaxon, Piperacillin/Tazobactam, Meropenem, Ciprofloxacin, Clarithromycin, Clindamycin, Vancomycin; Aciclovir
Immunosuppressive treatment	Dexamethasone, Hydrocortisone (in late stage of disease)

*Bacterial, mycobacterial, and fungal cultures from CSF and the biopsy yielded no growth.
†The patient exhibited IgG antibodies against adenoviruses, influenza viruses, enteroviruses, herpes simplex virus, Epstein-Barr virus, varicella-zoster virus, measles virus, and rubella virus, however, without IgM antibodies.
‡Elevated anti-recoverin antibodies were detected by immunoblot postmortem.

Technical Appendix Table 2. Real-time RT-PCR results for VSBV-1 of samples from the deceased encephalitis patient from the zoo*

Sample	Qualitative detection of VSBV-1 RNA/Quantification cycle (Cq-value)
CSF	positive/29.7
Lateral ventricle	positive/35.2
Lateral ventricle, plexus 1	positive/24.5
Lateral ventricle, plexus 2	positive/24.1
Striatum	positive/32.6
Substantia nigra	positive/27.9
Cerebellum	positive/32.7
Cortex frontal 1	negative/no Cq-value
Cortex frontal 2 (biopsy specimen)	negative/no Cq-value
Cortex temporo-lateral 1	negative/no Cq-value
Cortex temporo-lateral 2	negative/no Cq-value
Cortex parietal 1	negative/no Cq-value
Cortex parietal 2	negative/no Cq-value
Cortex occipital 1	negative/no Cq-value
Cortex occipital 2	negative/no Cq-value
Myocardium	negative/no Cq-value
Lung	negative/no Cq-value
Kidney	negative/no Cq-value
Liver	negative/no Cq-value
Spleen	negative/no Cq-value
Pancreas	negative/no Cq-value
Bone marrow	negative/no Cq-value
Intestine	negative/no Cq-value

*All tissue samples were formalin-fixed paraffin-embedded tissues. In FFPE samples, the analytical sensitivity of the PCR is often decreased (~10 Cq values). FFPE, formalin-fixed paraffin-embedded.

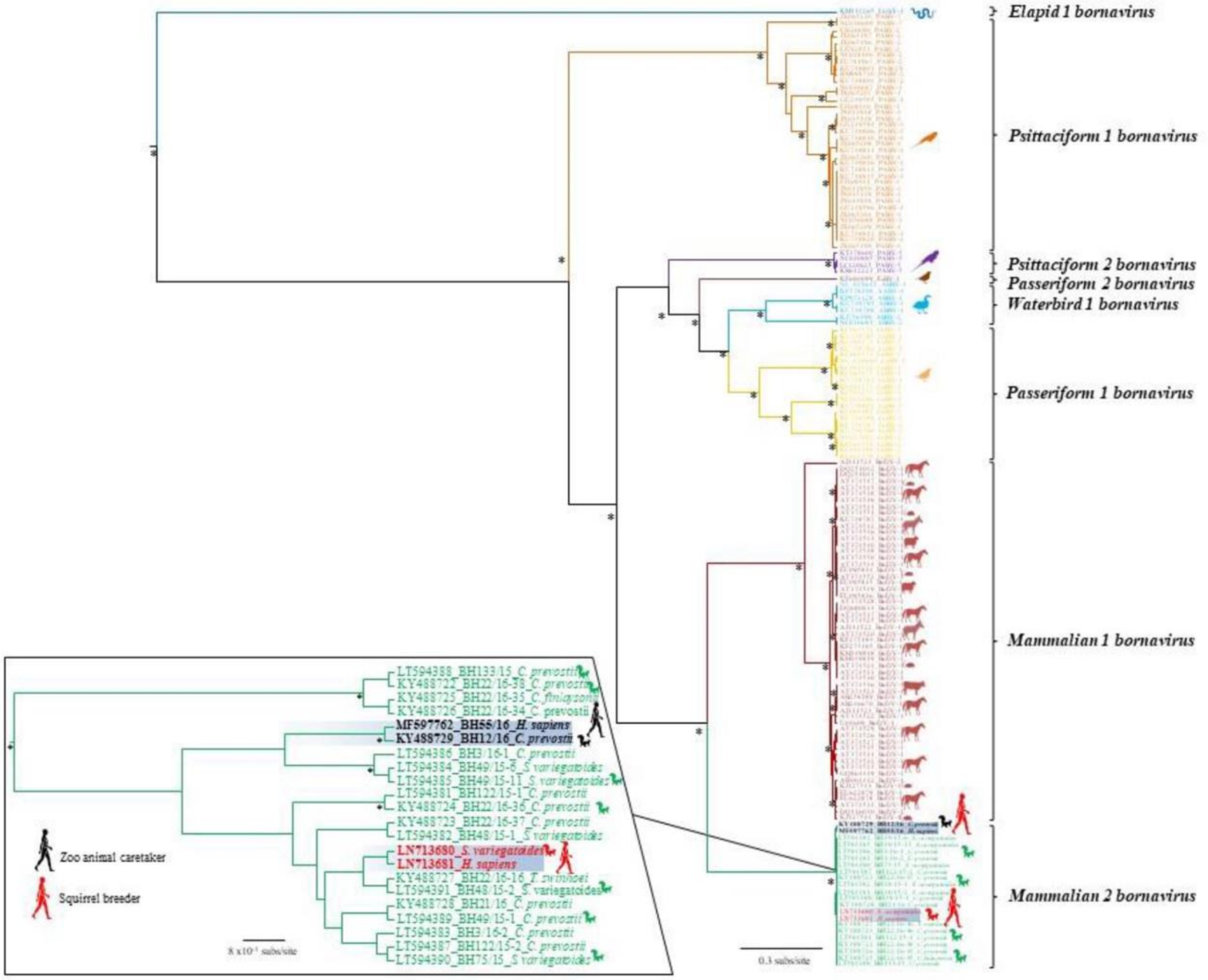

Technical Appendix Figure 1. Phylogenetic analysis of N protein (p40) gene sequences of *Mammalian 2 bornavirus* strains and other members of the *Bornaviridae*. The phylogenetic tree was inferred by using the Bayesian Markov Chain Monte Carlo method and in parallel a maximum-likelihood (ML) method (tree not shown). Statistical support of grouping from Bayesian posterior probabilities (clade credibilities ≥90%) and ML bootstrap replicates (≥70%) are indicated with an asterisk. Taxon information includes GenBank accession number, virus abbreviation, and year of detection. The *Mammalian 2 bornavirus* sequences generated during this study are bolded (black marked) and the host species from which the virus was isolated/detected is also provided. For better visualization of the phylogenetic relationship of the *Mammalian 2 bornavirus* strains detected in the zoo keeper and the contact squirrel, we magnified the complete *Mammalian 2 bornavirus* lineage which indicates the locations of both strains in the phylogeny. Branches are colored based on bornavirus species. The scale bar represents nucleotide substitutions per site.

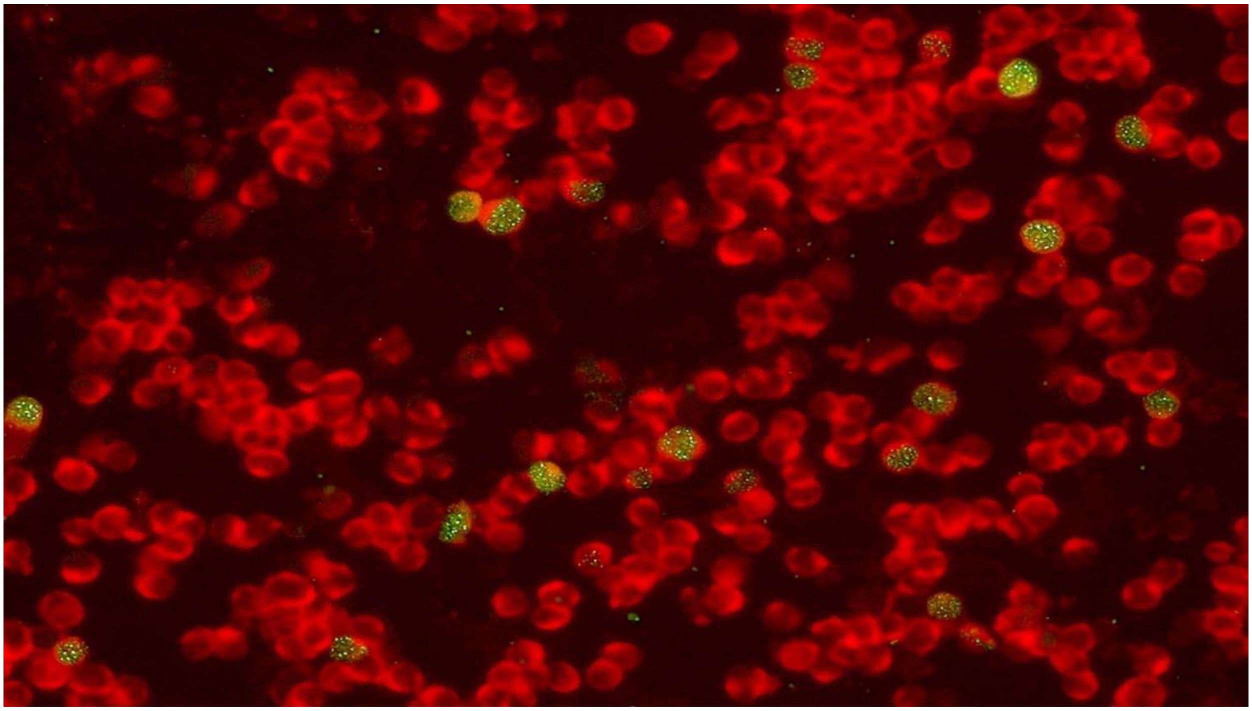

Technical Appendix Figure 2. Indirect immunofluorescence assay using the feline kidney cell line CRFK 227 persistently infected with *Mammalian 1 bornavirus* (BoDV). The patient's cerebrospinal fluid (CSF) showed a distinctive nuclear staining pattern, exhibiting an endpoint titer of 1:2,560 for IgG. CSF dilution 1:200, FITC-conjugated secondary goat-anti-human IgG antibody (Sifin Diagnostics, Berlin, Germany), 1:350.
